# Clinicopathological Characteristics of Breast Ductal Carcinoma In Situ: An Analysis of Chinese Population of 617 Patients

**DOI:** 10.1155/2021/8854418

**Published:** 2021-01-05

**Authors:** Guangmin Mao, Xiu-hua Shi, Xiaofang Wang, Xiaomeng Zhang, Xingxing Chen, Jinli Ma, Xiaoli Yu, Zhen Zhang, Xiaomao Guo

**Affiliations:** ^1^Department of Radiation Oncology, Fudan University Shanghai Cancer Center, Fudan University, Shanghai 200032, China; ^2^Department of Oncology, Shanghai Medical College, Fudan University, Shanghai, China; ^3^Department of Radiation Oncology, Longhua Hospital Shanghai University of Traditional Chinese Medicine, Shanghai 200032, China; ^4^Department of Radiation Oncology, The No. 2 People's Hospital of Wuhu City, Wuhu, Anhui Province, China

## Abstract

**Background:**

The purpose of this study was to describe the clinicopathological characteristics of breast DCIS in Chinese women and compare with that of patients in western countries.

**Method:**

From December 2005 to December 2015, 617 women diagnosed with pure DCIS after surgery at our institution were enrolled, and the clinicopathological characteristics were described.

**Results:**

In this study, the percentage of patients detected on screening, diagnosed at ≤50 years of age, with tumor size ≤2.0 cm, and with low-intermediate grade was 39.4%, 56.7%, 72.6%, and 77.4%, respectively, as compared to 50–80%, 20–30%, 70–90%, and 40–60% in published reports from western countries. The percentage of ER-positive patients was 76.3% in this study, which is similar to the mean expression rate of ER (mean: 68.7%, range: 49–96.6%) reported previously.

**Conclusions:**

The clinicopathological characteristics of Chinese DCIS patients include less detection on screening, younger age at diagnosis, and more low-intermediate nuclear grade.

## 1. Introduction

With the introduction of mammography screening, the incidence of ductal carcinoma in situ (DCIS) of breast has increased dramatically in last decades and now comprises 20–25% of all detected breast cancers [1, 2]. Although it is uncertain what exact rate of DCIS will progress to invasive cancer, 14–53% of the DCIS patients who were misdiagnosed as benign lesions turned out to be invasive breast cancer 10 years later [3]. Clinicopathological factors, such as age, estrogen receptor (ER) and/or progesterone receptor (PR) status, nuclear grade, and tumor size, were believed to be associated with risk of local recurrence (LR) and progression to invasive cancer in DCIS patients [4, 5]. It was reported that clinicopathological characteristics of invasive breast cancer patients in China differ from that of patients from western countries [6], which reminded us that the characteristics of Chinese DCIS patients may be unique as well. No large-scale population-based study on the clinicopathological characteristics of Chinese DCIS patients has been reported previously.

The purpose of this study was to describe the clinicopathological characteristics of breast DCIS in Chinese women and compare with that of patients in western countries.

## 2. Methods

### 2.1. Study Population

Of all the breast cancer patients diagnosed at our institute from December 2005 to December 2015, 1392 patients were identified to have breast DCIS after surgery. Patients who were male (*n* = 1), or with DCIS with microinvasion (*n* = 271), or diagnosed with invasive breast cancer after further surgery (*n* = 417) were excluded. Only primary pure DCIS patients were included in our study. All the primary pure DCIS diagnosis and pathological characteristics were confirmed by at least two pathologists based on surgical specimens. We also excluded 86 patients either for the history of previous invasive breast cancer or lack of information. The final cohort included 617 patients.

### 2.2. Clinicopathological Characteristics

Clinical characteristics, such as the detection method and age at initial diagnosis, were collected. The detection method referred to screening-detected by imaging (e.g., ultrasonography and mammography) and clinical symptoms (e.g., a noticed lump, nipple discharge, and breast pain).

Pathological characteristics including the tumor size, nuclear grade, ER status, PR status, and surgical margins were extracted from the original pathological reports based on surgical specimens. The tumor size was defined as the largest histologic dimension, including any discontinuous areas (e.g., multifocal lesions). The nuclear grade was pathologically assessed and classified as “low,” “intermediate,” and “high.” Low/intermediate grade was defined by the presence of nuclear grades 1 or 2 with limited or no foci of necrosis. High grade was defined by the presence of nuclear grade 3 atypia and comedo-type necrosis that was zonal (i.e., present in contiguous ductal spaces).

ER status and PR status were evaluated by immunohistochemistry (IHC) staining. A 1% cutoff value was used to dichotomise cases into positive and negative [7]. The surgical margin was pathologically assessed and classified as “negative” with margin width ≥2 mm, “close” with margin width between 0 and 2 mm, and positive.

### 2.3. Definition of Patient Subgroups

Patient subgroups were defined according to the detection method, age at diagnosis, tumor size, nuclear grade, and hormone receptor status.

Patients detected by imaging at time of screening or incidentally found in tissue of an otherwise benign biopsy were classified as the “screening” group, and patients who presented with clinical signs at diagnosis were classified as the “symptomatic” group. A cutoff value of 50 years for age at initial diagnosis was used to define age subgroups (≤50 vs >50), and a cutoff value of 2.0 cm was used to define subgroups for tumor size (≤2.0 cm vs >2.0 cm). Patients with low-grade, low-intermediate grade, or intermediate grade DCIS were categorized as the “low-intermediate grade” group, whereas patients with intermediate-high grade or high-grade DCIS were categorized as the “high-grade” group.

## 3. Statistical Analysis

Descriptive statistics were used to analyze the clinicopathological characteristics of Chinese DCIS patients. All statistical analyses involved in this study were performed using SPSS Version 22.0 (IBM Corporation, Armonk, NY, USA).

## 4. Results

The study population consisted of 617 women who presented with pure DCIS and was treated at our institute. Of them, 470 patients (76.2%) underwent total mastectomy, and the rest underwent breast-conserving surgery (BCS) with (*n* = 106) or without (*n* = 41) adjuvant radiotherapy. Following local therapy, 328 patients (53.2%) received adjuvant endocrine therapy. Patients' clinicopathological characteristics are listed in Table 1.

Of all the patients, 243 (39.4%) were categorized in the “screening” group, and 362 (58.7%) were categorized in the “symptomatic” group. The median age at the time of initial diagnosis was 48 years (range: 19–88 years). Using 50 years of age at initial diagnosis as a cutoff value, 350 patients (56.7%) were classified as the “≤50 years” group, and 267 patients (43.3%) were classified as the “>50 years” group. 606 patients (98.2%) had unilateral disease, and 601 patients (97.4%) presented with unicentric lesion. The tumor size was measuring ≤2.0 cm in 448 cases (72.6%), 2.1–5.0 cm in 120 patients (19.4%), and >5 cm in 6 patients (9.7%). The nuclear grade was “low-intermediate” in 463 patients (75.0%) and “high” in 135 patients (21.9%). On IHC staining, ER was positive in 471 patients (76.3%) and PR positive in 426 patients (69.0%).

Table 2 shows comparison of clinicopathological characteristics of patients with pure DCIS between this and previous studies.

Table 2 compared our data with published data from western countries by the year of 2015 [8–17]. In brief, the comparison showed that the percentage of patients detected on screening was only 39.4% in our study, as compared to 50% to 80% in previous studies; the percentage of patients with age≤50 years in our study was around 20% higher than that reported in other studies. The percentages of patients with tumor size ≤2.0 cm were 78.1%, which is similar to findings from SEER study, but is 10% lower than that reported by other studies including NSABP B-24 and EORTC. The percentage of patients with low-intermediate grade disease was 77.4% in this study, which is around 10%–40% higher than that reported in other studies. Great variations were observed to exist in the rates of ER and PR expression among studies.

## 5. Discussion

Previous data demonstrated that striking differences existed in the incidence rates and clinicopathological features of invasive breast cancer among women from diverse countries and different ethnic and genetic backgrounds [6]. However, it is not clear whether a similar situation exists for noninvasive breast cancer. The current study analyzed the clinicopathological characteristics of 617 Chinese women with pure DCIS, in comparison with published data of patients from western countries. To our knowledge, this is so far the first population-based study dealing with the differences in clinicopathological features of noninvasive breast cancer among women from China and western countries.

### 5.1. Detection Method

A significant negative association was reported to exist between screening-detected DCIS and subsequent invasive interval cancers, and the detection and treatment of DCIS was considered to be worthwhile in prevention of future invasive disease [18]. The DCIS with clinical symptoms was believed to be biologically different from screening-detected DCIS, and the clinical symptoms might be a reaction to“early” invasive breast cancer in tumor microenvironment [5]. The detection method was related to the LR rate in pure DCIS cases.

The percentage of screening-detected patients in our study was much lower than that in published data. Factors that were associated with lower rates of screening might include not being covered by medical insurance, lack of screening awareness, fear of mammography X-ray, and inadequate system level interventions. To enhance the screening rates, efforts should be made to promote breast health education, publicize the breast cancer screening guideline, and increase access to breast cancer screening services among low-income populations by enrolling in low-cost or free mammography programs.

### 5.2. Age at Diagnosis

The age at diagnosis was proven to be an independent prognostic factor for LR in patients with pure DCIS after BCS and was combined with other factors by Van Nuys Prognostic Index (VNPI) to predict for risk of LR [19]. Young age, especially ≤40–50 years, was associated with a higher risk of LR. The percentage of patients with age ≤50 y in our study was around 20% higher than that reported in previous studies. It is unknown whether the difference in the percentage of patients aged ≤50 y was associated with a difference in the LR rate between China and western countries. The outcome of ongoing follow-up of this cohort of patients will help to answer this question.

### 5.3. Tumor Size and Nuclear Grade

The tumor size and nuclear grade were reported to be independent prognostic factors that predicted risk of LR among patients with pure DCIS [20, 21]. Generally, tumor size ≤1.5–2.5 cm and low-intermediate grade represents lower risk of LR [22]. The percentage of patients with tumor size ≤2.0 cm was 72.6% in this study, which is similar to findings from SEER study [9] but is 10% lower than that reported by other studies including NSABP B-24 [16, 17] and EORTC [15]. The percentage of patients with low-intermediate grade was 77.4%, which is significantly higher than that reported in other studies listed in Table 2.

The RTOG 9804 randomized clinical trial included women with screen-detected DCIS, ≤2.5 cm size, low to intermediate nuclear grade, resected with margins negative at ≥3 mm and observed an IBTR risk of 6.7% in the observation arm, compared to 0.9% in the whole breast irradiation (WBI) arm at a median follow-up of 7.2 years [23]. Similar results were noted in the initial publication of ECOG 5194 trial among patients meeting similar criteria [24]. These inclusion criteria therefore define a group of patients with low-risk DCIS for whom observation confers a low absolute risk of IBTR and for whom the addition of WBI confers a small but measurable absolute benefit in prevention of IBTR.

### 5.4. Status of Hormone Receptors

The status of hormone receptors (HRs) including ER and/or PR was an independent predictor of the LR risk as well as effect of endocrine therapy. In this study, 76.3% of patients were ER positive and 69.0% were PR positive, which is comparable to the mean rate of ER expression (mean: 68.7%, range: 49–96.6%), as reported in a systemic review by Lari and Kuerer from the University of Texas MD Anderson Cancer Center [25]. Similar finding was noted for PR expression. Generally, following local therapy for pure DCIS, 5 years of endocrine therapy with either tamoxifen or an aromatase inhibitor is recommended for ER- and/or PR-positive patients to reduce the risk of ipsilateral breast events and contralateral breast cancer.

### 5.5. Adjuvant Radiotherapy and Endocrine Therapy

Following BCS, whole breast irradiation (WBI) with or without subsequent boost to tumor bed was reported to reduce 12.0% absolute risk and 37.5% relative risk in ipsilateral breast events at 20 years [26]. In this study, the percentage of patients receiving BCS with or without adjuvant radiotherapy was relatively low, as compared to 1991–2010 SEER data. Efforts should be done to promote the shift of treatment patterns from mastectomy to BCS, as well as the shift from WBI to accelerated partial breast irradiation (APBI) for low-risk DCIS patients who meet abovementioned criteria, according to 2016 ASTRO guideline [27].

Although ER- and/or PR-positive DCIS patients are mandated to receive endocrine therapy by NCCN guidelines [28], only 69.6% of HR-positive patients received tamoxifen, toremifene, or aromatase inhibitor in this study. This finding is similar to reports from western countries.

## 6. Conclusions

Compared to published data from western countries, the clinicopathological characteristics of Chinese DCIS patients include the following: (1) lower percentage of patients detected on screening, (2) higher percentage of patients under age of 50 years at diagnosis, and (3) higher percentage of patients with low-intermediate nuclear grade DCIS. Further analysis is warranted to be done to determine whether Chinese DCIS patients have a better prognosis than the patients from western countries.

## Figures and Tables

**Table 1:** Clinicopathological characteristics of Chinese DCIS patients (*n* = 617).

Characteristics	No. of cases	%
Detection method		
Symptomatic	362	58.7
Screening	243	39.4
NA	12	1.9
Age (years)		
Median	48	
≤40	122	19.8
41–50	228	37.0
51–60	151	24.5
61–70	81	13.1
＞70	35	5.7
Tumor size (cm)		
≤1.0	213	34.5
1.1–2.0	235	38.1
2.1–5.0	120	19.4
＞5.0	6	9.7
NA	43	7.0
Nuclear grade		
Low-intermediate	463	75.0
High	135	21.9
NA	19	3.1
ER status		
Positive	471	76.3
Negative	119	19.3
NA	27	4.4
PR status		
Positive	426	69.0
Negative	168	27.2
NA	23	3.7

DCIS: ductal carcinoma in situ; NA: not available; ER: estrogen receptor; PR: progesterone receptor; IHC: immunohistochemistry.

**Table 2:** Comparison of clinicopathological characteristics of patients with pure DCIS between this and published studies.

Characteristics	Subgroup	Our study	SEER 2015	NSABP-B24	EORTC	SweDCIS	B17	Canada	Nottingham 2019	UCSD 2020
*Year of research*	—	2005–2015	1988–2011	1986–2009	1986–1996	1987–1999	1985–1990	1994–2003	1990–2012	2010–2016

*No. of patients*	—	617	108.196	2.061	1010	1046	790	571	651	1540

*Detection method*	Symptomatic	58.7%	—	8.7%	27.2%	27.6%	8.1%	—	48%	—
	Screening	39.4%	—	82.6%	71.6%	72.4%	80.8%	—	52%	—

*Age (years)*	≤50 y	56.7%	33.9%	33.5%	—	24.1%	33.3%	19.3%	—	—
	＞50 y	43.3%	66.1%	66.5%	—	75.9%	66.7%	80.7%	—	—

*Tumor size (cm)*	≤2.0 cm	78.1%	77.0%	94.2%	91.2%	67.5%	87.8%	—	—	—
	＞2.0 cm	21.9%	23.0%	4.3%	8.8%	21.8%	8.9%	—	—	—

*Nuclear grade*	Low-intermediate	77.4%	54.0%	54.0%	62.3%	50.2%	—	67.7%	39.2%	57.3%
	High	20.9%	46.0%	42.3%	37.7%	49.8%	—	32.3%	60.8%	42.7%

*ER status*	Positive	76.3%	42.5%	28.6%	52.7%	—	—	94.7%	75.3%	84.2%
	Negative	19.3%	8.2%	8.3%	19.8%	—	—	5.3%	24.7%	15.5%
	NA	4.4%	49.2%	63.1%	27.5%	—	—	—	—	0.3%

*PR status*	Positive	69.0%	—	—	44.3%	—	—	—	46.7%	74.2%
	Negative	26.3%	—	—	23.1%	—	—	—	53.3%	25.2%
	NA	3.7%	—	—	32.7%	—	—	—	—	0.6%

SEER: surveillance, epidemiology, and end results; NSABP: National Surgical Adjuvant Breast and Bowel Project; EORTC: European Organisation for Research and Treatment of Cancer; SweDCIS: Swedish ductal carcinoma in situ; UCSD: University of California, San Diego; other abbreviations as in Table 1.

## Data Availability

The data used to support the findings of this study are available from the corresponding author upon request.
